# Characterization of Single-Chain Fv Fragments of Neutralizing Antibodies to Rabies Virus Glycoprotein

**DOI:** 10.3390/v13112311

**Published:** 2021-11-19

**Authors:** Kohei Yumoto, Tomoaki Arisaka, Kazuma Okada, Kyosuke Aoki, Toyoyuki Ose, Tatsunori Masatani, Makoto Sugiyama, Naoto Ito, Hideo Fukuhara, Katsumi Maenaka

**Affiliations:** 1 Laboratory of Biomolecular Science, Faculty of Pharmaceutical Sciences, Hokkaido University, Sapporo 060-0812, Japan; yuko-9-jud-6@pharm.hokudai.ac.jp (K.Y.); arisaka@toyoreizo.co.jp (T.A.); shishiroku624@gmail.com (K.A.); ose.toyoyuki@sci.hokudai.ac.jp (T.O.); 2 Faculty of Applied Biological Sciences, Gifu University, Gifu 501-1193, Japan; okada@iph.osaka.jp (K.O.); mstn@gifu-u.ac.jp (T.M.); sugiyama@gifu-u.ac.jp (M.S.); naotoito@gifu-u.ac.jp (N.I.); 3 Center for Research and Education on Drug Discovery, Hokkaido University, Sapporo 060-0812, Japan; h-fukuhara@pharm.hokudai.ac.jp; 4 Global Station for Biosurfaces and Drug Discovery, Faculty of Pharmaceutical Sciences, Hokkaido University, Sapporo 060-0812, Japan

**Keywords:** rabies virus, rabies virus glycoprotein, single-chain Fv, Fab, neutralization antibody, biolayer interferometry (BLI), differential scanning fluorometry (DSF)

## Abstract

Rabies has almost a 100% case-fatality rate and kills more than 59,000 people annually around the world. There is no established treatment for rabies. The rabies virus (RABV) expresses only the glycoprotein (RABVG) at the viral surface, and it is the target for the neutralizing antibodies. We previously established mouse monoclonal antibodies, 15–13 and 12–22, which showed neutralizing activity against the RABV, targeting the sequential and conformational epitopes on the RABVG, respectively. However, the molecular basis for the neutralizing activity of these antibodies is not yet fully understood. In this study, we evaluated the binding characteristics of the Fab fragments of the 15–13 and 12–22 antibodies. The recombinant RABVG protein, in prefusion form for the binding analysis, was prepared by the silkworm–baculovirus expression system. Biolayer interferometry (BLI) analysis indicated that the 15–13 Fab interacts with the RABVG, with a *K*_D_ value at the nM level, and that the 12–22 Fab has a weaker binding affinity (*K*_D_ ~ μM) with the RABVG compared to the 15–13 Fab. Furthermore, we determined the amino acid sequences of both the antibodies and the designed single-chain Fv fragments (scFvs) of the 15–13 and 12–22 antibodies as another potential biopharmaceutical for targeting rabies. The 15–13 and 12–22 scFvs were successfully prepared by the refolding method and were shown to interact with the RABVG at the nM level and the μM level of the *K*_D_, respectively. These binding characteristics were similar to that of each Fab. On the other hand, differential scanning fluorometry (DSF) revealed that the thermal stability of these scFvs decreases compared to their Fabs. While the improvement of the stability of scFvs will still be required, these results provide insights into the neutralizing activity and the potential therapeutic use of antibody fragments for RABV infection.

## 1. Introduction

The rabies virus (RABV) is a zoonotic virus that belongs to the *Lyssavirus* genus of the *Rabdoviridae* family and causes lethal neurological symptoms [1]. Infection by RABV is preventable by vaccination, and even if people are exposed to the virus by bite wound, postexposure prophylaxis (PEP) prevents the onset of disease in many cases [2]. Nevertheless, there are 59,000 human deaths annually, mainly in Asia and Africa, according to the World Health Organization (WHO) [3]. The reasons for this problem are thought to include the high cost of vaccines, poor control of dog rabies, transportation-to-treatment problems, and a large lack of political will, which has suppressed an increase in vaccine coverage in developing countries [4,5]. In addition, there is still no established treatment after the onset of the disease.

RABV is an RNA virus with a negative-strand RNA genome, which encodes genes for nucleo- (N) protein, RNA polymerase component (L), matrix (M) protein, phospho- (P) protein, and glyco- (G) protein. Rabies virus glycoprotein (RABVG) is the trimer protein expressed on the surface of the envelope that binds to the host cell receptors (nicotinic acetylcholine receptor (nAchR), neural cell adhesion molecule (NCAM), low-affinity nerve growth factor receptor (p75NTR)) by its extracellular domain to achieve the infection [6,7,8,9]. In addition, RABVG also has a role in the membrane fusion with the host cells and is thought to be the virulence factor [10,11]. RABVG is the major antigen that induces the neutralizing antibody to suppress the infection with RABV. We previously established 35 types of mouse anti-RABVG monoclonal antibodies and analyzed the antigenic structure and biological properties of the RABVG [12]. However, the molecular basis for the neutralizing activity of these antibodies is yet to be fully understood. In addition, their antibody fragments, potentially useful for the cost and efficiency of medical treatment, have not yet been evaluated.

In this study, we cloned and determined the amino acid sequences of two anti-RABVG antibodies, 15–13 and 12–22, which were established in a previous report as described above [12], and which showed neutralizing activity against multiple strains, including street viruses. We prepared Fab fragments of the 15–13 and 12–22 antibodies, which recognize the sequential and conformational epitopes on the RABVG, respectively [12], to evaluate their RABVG-binding characteristics. Furthermore, we also designed and analyzed the single-chain Fv fragments (scFvs) of the 15–13 and 12–22 antibodies as other potential biopharmaceuticals for targeting rabies. The thermal stability of these antibody fragments was measured by differential scanning fluorometry (DSF). The recombinant RABVG was prepared by the silkworm–baculovirus expression system and was used for the evaluation of the binding properties with these antibody fragments using biolayer interferometry (BLI) analysis. The 15–13 scFv exhibited high affinity (*K*_D_ ~ nM), and the 12–22 scFv exhibited medium affinity (*K*_D_ ~ μM), essentially the same as those of their Fab constructs. These results provide important insights into the neutralizing activity and potential therapeutic use of antibody fragments for RABV infection.

## 2. Materials and Methods

### 2.1. Construction of Expression Plasmid for 15–13 and 12–22 scFvs

The total RNA was extracted from mAb-producing hybridoma cells, which has previously been established [12]. The cDNA was reverse transcribed with the RT-PCR kit (Invitrogen, Carlsbad, CA, USA) using oligo dT primer. The variable domains of heavy-chain (VH) and light-chain (VL) genes were separately amplified by PCR reactions with primer mixture sets without linker sequences, as described in the paper of Imai et al. [13], and were inserted into pGEM-T Easy vector (Promega, Madison, WI, USA). The obtained gene was used as a template to construct the expression plasmid for scFvs, with both the VH–VL and VL–VH orientations, scFv HL and scFv LH, respectively. The splicing by the overlap extension PCR method via the sequence encoding the glycine–serine (GGGGS)_3_ flexible linker was applied with primer pairs, summarized in Table 1. The PCR was performed using KOD-Plus DNA polymerase (TOYOBO, Osaka, Japan) according to the manufacturer’s instructions. The amplified PCR products were digested by the restriction enzymes, *Nde* I and *Eco* RI, and ligated into pET−22b(+) vector (Novagen) between the *Nde* I and *Eco* RI of the multiple cloning site.

### 2.2. Expression and Purification of 15–13 and 12–22 scFvs

The expression plasmid of each scFv was transformed into the *Escherichia coli* strain, BL21-CodonPlus (DE3)-RIL. The bacterial cells were grown in 2xYT medium, supplemented with 100 μg/mL ampicillin at 37 °C, to an optical density at 600 nm (OD_600_) of 0.5. Expression was induced with 1 mM isopropyl-β-D-thiogalactopyranoside (IPTG) at 37 ^o^C for 4 h. The scFv expressed as inclusion bodies was washed, solubilized with guanidine hydrochloride buffer (50 mM Tris-HCl pH8.0, 6 M guanidine HCl, 10 mM EDTA), and refolded for 3 days using the dilution method previously reported. The refolded scFv was purified by size exclusion chromatography using a HiLoad 26/600 Superdex 75 column (GE Healthcare) with SEC buffer (50 mM MES pH6.0, 500 mM NaCl). For the biolayer interferometry analysis, the buffer was exchanged to Tris buffer (20 mM Tris-HCl pH8.0, 150 mM NaCl) using Amicon Ultra (Merck Millipore, Burlington, MA, USA).

### 2.3. Preparation and Purification of 15–13 and 12–22 Fab Fragments

mAb 15–13 and mAb 12–22 were purified from hybridoma-injected mouse ascites. The MAbs were precipitated by 50% saturated ammonium sulfate and were subsequently purified by Protein G Sepharose 4 Fast Flow (GE Healthcare, Chicago, IL, USA). The purified MAbs were digested by incubating them with immobilized papain (Thermo Fisher Scientific, Waltham, MA, USA) overnight, according to the manufacturer’s instructions. The digested samples were separated from the immobilized papain using a resin separator. The Fab fragments were purified by Protein A Sepharose Fast Flow (GE Healthcare), and anion exchange chromatography using Resource Q, 1 mL (GE Healthcare).

### 2.4. Preparation of Recombinant RABVG by Silkworm–Baculovirus Expression System

The gene-encoding extracellular domain of the RABVG (CVS strain), with a T4 fibritin trimerization domain and a hexahistidine tag in tandem at the C-terminal site, was ligated into the pFastBac1 vector (Invitrogen). To obtain the stable recombinant protein, two fusion loops (Asn95-Thr101 and Pro139-Val147) were substituted to the Gly–Gly–Ser–Gly–Gly linkers, using the primer set listed in Table 2. The subcloned pFastBac1 plasmid was introduced into the *E. coli* BmDH10Bac strain to generate a recombinant bacmid. The purified bacmid DNA (20 ng/μL) was mixed with DMRIE-C reagent (Invitrogen) and incubated at room temperature for 40 min. The bacmid DNA mixture was injected into fifth instar silkworm larvae, purchased from Ehime-Sanshu (Ehime, Japan). Silkworm was cultivated with an artificial feed (Nosan Corporation, Kanagawa, Japan) at 25 °C in an incubator. Five days after injection, the hemolymphs of the silkworm larvae were collected and 0.5% sodium thiosulfate was added to avoid melanization. The hemolymph was centrifuged at 5000× *g* for 10 min to remove cell debris. The supernatant was ultracentrifuged at 40,000× *g* for 30 min to precipitate the recombinant virus. The viral pellet was resuspended in 1 mL of PBS per larva and used for the subsequent protein expression. The supernatant containing the recombinant RABVG (rRABVG) was purified by 30% saturated ammonium sulfate precipitation. The precipitated sample was resuspended in resuspension buffer (20 mM Tris-HCl pH8.0, 150 mM NaCl, 1% (*w*/*v*) CHAPS) and purified by 5mL HisTrap FF (GE Healthcare) and Superdex 200 10/300 GL (GE Healthcare).

### 2.5. Thermal Stability Analysis by Differential Scanning Fluorometry (DSF)

The thermal stability of the Fab and scFv was evaluated by differential scanning fluorometry (DSF) using the CFX96 Touch qPCR System (Bio-Rad, Hercules, CA, USA), as follows. All analyses were performed in running buffer (20 mM Tris-HCl pH8.0, 150 mM NaCl). Thirty microliters of 2 μM protein solution was mixed with 2 μL of SYPRO orange (Invitrogen) protein dye (final 1:500 dilution). After an initial incubation at 20 °C for 2 min, the protein solution was heated from 20 °C to 95 °C, at scanning rates of 0.5 °C/30 sec, and the fluorescence was measured at each degree by the HEX channel. Data were analyzed using the Bio-Rad CFX manager, version 2.1. The melting temperature (*T_m_*) of each antibody fragment was calculated from the curve minim of the plot of derivative fluorescent-based signals against temperature.

### 2.6. Interaction Analysis Using Biolayer Interferometry (BLI)

Kinetic analyses of rRABVG were performed using a BLItz instrument (ForteBio, Fremont, CA, USA). Analyses were carried out by the advanced kinetics mode at room temperature, with shaking at 1000 rpm. Prior to the measurements, the Ni-NTA (NTA) biosensors (ForteBio) were soaked in running buffer (20 mM Tris-HCl pH8.0, 150 mM NaCl) for more than 10 min for hydration. The rRABVG, at a concentration of 0.1 mg/mL, was immobilized on a biosensor in the running buffer for 120 sec. The ligand-immobilized biosensors were transferred into solutions containing the serially twofold-diluted scFv or Fab in the running buffer to monitor association. The dissociation step was monitored by soaking the biosensors in the running buffer again. The association step was monitored for 120 sec. The dissociation step was monitored for 300 sec for 15–13 fragments, and for 120 sec for 12–22 fragments, respectively. To subtract the effects of the baseline drift, reference runs where the mAb was omitted at the binding step were performed. The binding curves were fitted in a 1:1 binding model, and the affinity constants (*K*_D_), and the association (*k*_on_) and dissociation (*k*_off_) rate constants were calculated by BLItz Pro version 1.2.1.3.

## 3. Results

### 3.1. Cloning of Variable Regions of 15–13 and 12–22 Antibodies and Preparation of Their Single-Chain Fv Fragments

**Figure 1:**
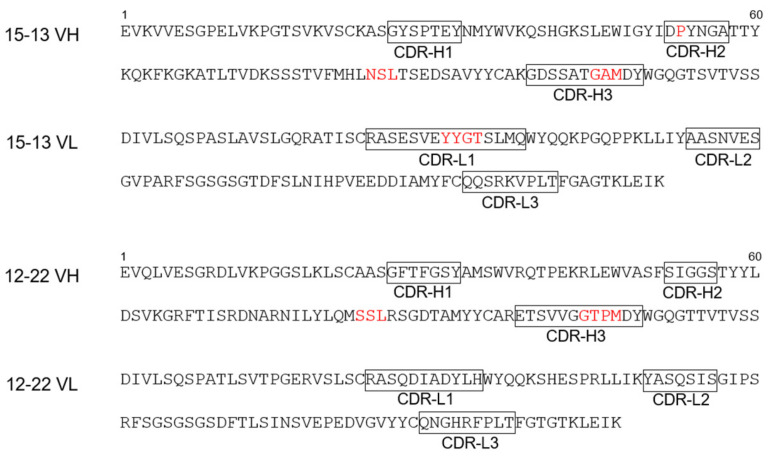
Sequence analysis of antibodies. The amino acid sequence of the VH and VL domains of 15–13 and 12–22 mAbs. CDRs are enclosed in a square. Red letters indicate the insertion of amino acid residues.

In order to determine the amino acid sequences of the variable regions of the heavy and light chains (VH and VL) of the 15–13 and 12–22 antibodies, the mRNAs were extracted from the hybridoma cells of each mAb. Using the primer sets suitable for the cloning of the mouse antibody variable regions [13], the mRNAs were used as the templates of the RT-PCR, and the genes encoding their variable regions were amplified. The determined sequences of the VH and VL encode 120 and 111 residues for the 15–13, and 120 and 107 residues for the 12–22, respectively (Figure 1). The complementarity determining regions (CDRs) for both the VH and VL were estimated according to the Chothia numbering scheme [14,15]. The 15–13 mAb have one (‘P’ as H52A) and three (‘GAM’ as H100A, H100B, and H100C) amino acid insertions in CDR-H2 and CDR-H3, respectively. In addition, the CDR-L1 of the 15–13 mAb has four amino acid insertions (‘YYGT’ as L30A, L30B, L30C, and L30D). On the other hand, the 12–22 mAb has four amino acids inserted in the CDR-H3 (‘GTPM’ as H110A, H100B, H100C, and H100D). Both the 15–13 and 12–22 mAb also have three amino acids inserted in the HFR3 (‘NSL’ and ‘SSL’ as H82A, H82B, and H82C). The obtained cDNAs were used as templates for cloning with the primer combinations listed in Table 1.

**Figure 2:**
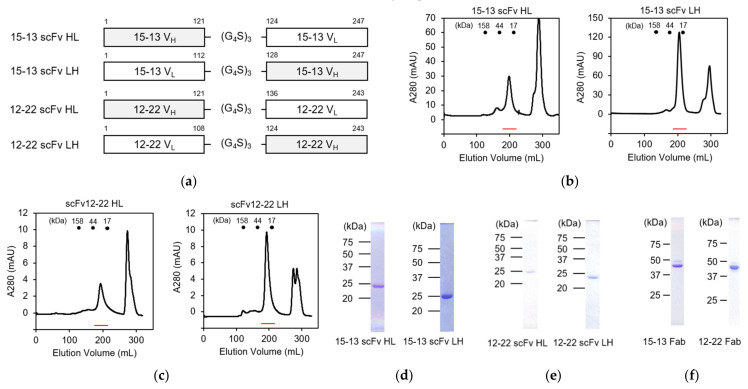
Construction and purification of anti-RABVG antibodies. (**a**) Schematic structures of 15–13 scFv HL and LH, and 12–22 scFv HL and LH; (**b**,**c**) The size exclusion chromatogram of refolded 15–13 scFv HL and LH (**b**), and 12–22 scFv HL and LH (**c**). Red lines represent the elution positions of scFvs; (**d**,**e**) SDS-PAGE of purified 15–13 scFv HL and LH (**d**), and 12–22 scFv HL and LH (**e**), under nonreducing conditions. (**f**) SDS-PAGE of purified 15–13 Fab and 12–22 Fab under nonreducing conditions.

For the preparation of scFvs, we initially designed the expression constructs of the 15–13 and 12–22 with different orders: VH-linker-VL (scFv HL) and VL-linker-VH (scFv LH) (Figure 2a). The VH and VL were connected by the (G_4_S)_3_ linker and cloned into the pET−22b(+) expression vector. All the constructs were expressed as inclusion bodies by *Escherichia coli* BL21-CodonPlus (DE3)-RIL, and refolded in vitro by a dilution method, in a similar way to the previous study [16]. The refolded scFvs were purified by size exclusion chromatography with a HiLoad 26/600 Superdex 75 column (Figure 2b,c). The elution profile and SDS-PAGE analysis (Figure 2d,e) indicated that these scFvs were highly purified as a monomer. The final yields for the scFv HL and the scFv LH of the 15–13 mAb are 5.3 and 12.4 mg/L of culture, respectively, indicating that the scFv LH is more efficiently expressed than the scFv HL (Table 3). On the other hand, the final yields for the scFv HL and the scFv LH of the 12–22 mAb are 0.8 and 2.2 mg/L of culture, respectively, indicating that the scFv LH is more efficiently expressed than the scFv HL (Table 3).

### 3.2. Preparation of Fab Fragments of 15–13 and 12–22 Antibodies

To compare the binding characteristics of the scFvs to RABVG, we also prepared Fab fragments of the 15–13 and 12–22 antibodies. The 15–13 and 12–22 mAbs were purified from hybridoma-injected mouse ascites by the ammonium sulfate precipitation method, with 50% ammonium sulfate, and a subsequent affinity chromatography with a Protein G column. The Fab fragmentation was performed using immobilized papain, and digestives were separated from beads by a resin separator. The Fc fragments were separated by affinity chromatography with Protein A Sepharose Fast Flow and anion exchange chromatography. The purity of the Fab fragments was confirmed by SDS-PAGE analysis (Figure 2f).

### 3.3. Construction and Purification of rRABVG

**Figure 3:**
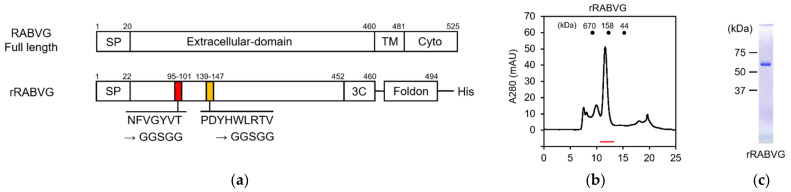
Construction and purification of rabies virus glycoprotein. (**a**) Schematic structures of the RABVG full length and the rRABGV. SP, signal peptide; TM, transmembrane domain; Cyto, cytoplasmic tail; 3C, HRV-3C protease cleavage site; Foldon, T4 fibritin trimerization domain. (**b**) The size exclusion chromatogram of rRABVG. The red line represents the elution position of rRABVG. (**c**) SDS-PAGE of purified rRABVG under reducing conditions.

To evaluate the binding of the scFvs with the RABVG by biolayer interferometry (BLI) analysis, we also prepared a recombinant extracellular domain of RABVG (hereafter designated as rRABVG) using the silkworm–baculovirus expression system. The C-terminal site of the rRABVG was fused with the T4 fibritin domain for stable expression [17]. In addition, the sequences presumed to the two fusion loops were replaced by the GGSGG linker to improve the properties of the rRABVG [11] (Figure 3a). The prepared recombinant baculovirus was injected into the silkworm. The rRABVG contained in the body fluids were fractionated with 30% saturated ammonium sulfate and subsequently purified by Ni^2+^ affinity chromatography and size exclusion chromatography, showing that the rRABVG formed a trimer with a single peak (Figure 3b). SDS-PAGE analysis indicated that the rRABVG was highly purified and suitable for further experiments (Figure 3c).

### 3.4. Differential Scanning Fluorimetry (DSF)

**Figure 4:**
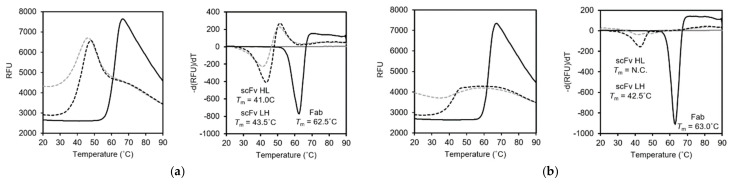
Thermal stability assay of scFvs and Fabs. (**a**,**b**) DSF analyses of 15–13 (**a**), and 12–22 (**b**). DSF plots (raw data, left) and plot of derivative fluorescent-based signal against temperature (right) are represented. Solid lines represent Fab fragment. Dotted lines represent scFv HL (gray) and LH (black). Tm values were measured from the curve minima. N.C. means “could not be calculated”.

To evaluate the thermal stabilities of the recombinant scFv and Fab fragments, we performed a differential scanning fluorometry (DSF) analysis. The melting temperature (*T*_m_) was calculated from the curve minim of the plot of the derivative fluorescent-based signals against temperature. The DSF analysis showed a *T*_m_ of 62.5 °C in the 15–13 Fab. On the other hand, the *T*_m_ of the15–13 scFv HL and LH were 41.0 °C and 43.5 °C, respectively (Figure 4a, right). This result suggests that scFv engineering reduced the stability. Next, the *T*_m_ of the 12–22 scFv LH was determined to be 42.5 °C. On the other hand, that of the 12–22 scFv HL could not (Figure 4b, right). The DSF plot of the 12–22 scFv HL showed that the RFU at 20 °C was higher than the others, and this is the reason why the minimum could not be obtained (Figure 4b, left). A similar tendency was observed for the 15–13 scFv HL (Figure 4a, left). Since SYPRO orange, a fluorescent dye used in this study, binds to the hydrophobic region of the protein, the hydrophobic part of both 15–13 and 12–22 is likely to be exposed and is relatively unstable by single-chain formation in the VH–VL order.

### 3.5. Biolayer Interferometry (BLI) Analysis of the rRABVG-scFv Interaction

**Figure 5:**
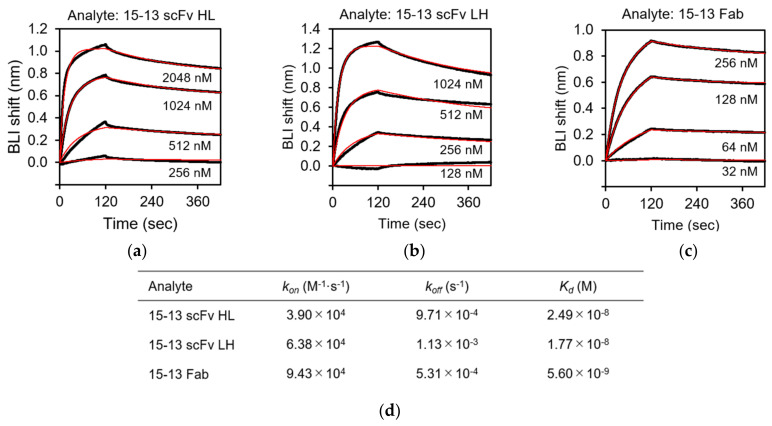
Binding analysis of rRABVG by 15–13 fragments. Binding analysis of anti-RABVG antibodies with rRABVG immobilized on the Ni-NTA biosensor were performed. Representative sensorgrams for the rRABVG binding responses of: (**a**) 15–13 scFv HL; (**b**) 15–13 scFv LH; and (**c**) 15–13 Fab are shown. The sensorgrams were globally fitted with a 1:1 binding model (red lines). (**d**) Affinity constants (*K*_d_), association (*k*_on_), and dissociation (*k*_off_) rate constants were derived by nonlinear curve fitting using BLItz Pro version 1.2.1.3.

**Figure 6:**
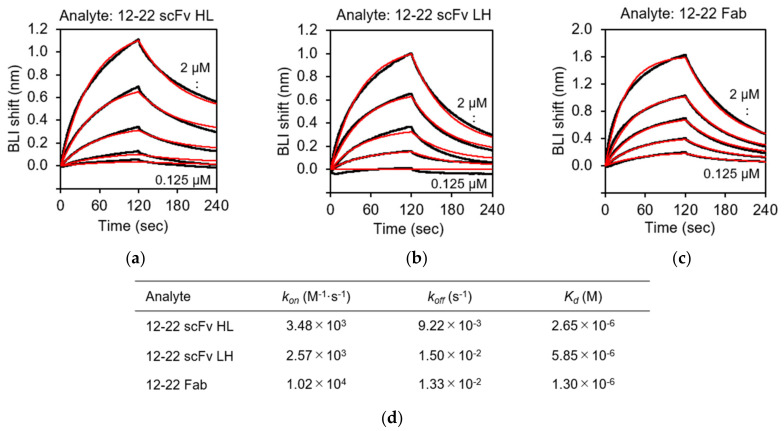
Binding analysis of rRABVG by 12–22 fragments. Representative sensorgrams for the binding responses between rRABVG and (**a**) 12–22 scFv HL; (**b**) 12–22 scFv LH; and (**c**) 12–22 Fab. Sensorgrams were globally fitted with 1:1 binding model (red lines). (**d**) Affinity constants (*K*_d_), association (*k*_on_), and dissociation (*k*_off_) rate constants were derived by nonlinear curve fitting using BLItz Pro version 1.2.1.3.

BLI analysis was carried out to characterize the recognition of rRABVG by the purified scFv and Fab fragments using the BLItz instrument. rRABVG was immobilized on the surface of the Ni-NTA biosensor, which easily captures His-tagged proteins. The immobilized biosensor was dipped in a solution of serially twofold-diluted scFv and Fab fragments, and the association was monitored. The dissociation was monitored when the biosensor was immersed in the buffer solution. The sensorgrams at each concentration were derived by subtracting the responses of the reference runs where the mAb immobilization step was omitted. The kinetic parameters of the bindings were calculated by the fitting with the 1:1 binding model. The 15–13 scFv HL, LH, and the Fab showed specific binding to the rRABVG, and their *K*_D_ values were determined to be 24.9, 17.7, and 5.60 nM, respectively (Figure 5a,d). The *k*_on_ and *k*_off_ values of the 15–13 scFv HL were 3.90 × 10^4^ (M^−1^·s^−1^) and 9.71 × 10^−4^ (s^−1^), respectively (Figure 5d). On the other hand, the *k*_on_ and *k*_off_ values of the 15–13 scFv LH were 6.38 × 10^4^ (M^−1^·s^−1^) and 1.13 × 10^−3^ (s^−1^), respectively (Figure 5d). These results indicated that both the 15–13 scFvs HL and LH showed slightly weaker binding affinities with rRABVG compared to the 15–13 Fab. The 12–22 scFv HL and LH and the Fab also showed specific binding to the rRABVG, with *K*_D_ values in the micromolar range; thus, the affinities were lower than those of the 15–13 fragments (Figure 6a,d). The kinetic analysis showed that faster dissociation and slower association contribute to lower affinities.

## 4. Discussion

The onset of rabies is prevented by pre-exposure or postexposure prophylaxis (PEP), using a combination of vaccine and polyclonal serum, and serum-derived human rabies immunoglobulin (HRIG). However, since the current use of HRIG is expensive, rabies still occurs mainly among low-income groups in Asia and Africa. In addition, the relatively short shelf life and the inclusion of non-neutralizing antibodies may affect efficacy because of their polyclonal characteristics [18]. For these reasons, the development of inexpensive antibody products that could be a substitute for HRIG is eagerly awaited. In 2018, the WHO recommended that monoclonal antibodies (mAb products) be used instead of HRIG if available [19]. Several antibody candidates have been identified as potential alternatives to HRIG, such as CL184 and SYN023, and clinical trials have been undergone [20,21,22]. In order to neutralize multiple strains of the RABV, almost all of them are antibody cocktails containing multiple mAbs that recognize different epitopes: CL184 cocktail is composed of two mAbs, CR57 and CR4098; and SYN023 is composed of CTB001 and CTB012 mAbs.

We previously established 35 types of anti-RABVG mouse monoclonal antibodies for analyzing the antigenic structure and biological properties of RABV [12]. Here, we constructed and evaluated the binding and neutralizing characteristics of the anti-RABVG antibody fragments, Fab and scFvs, of 15–13 and 12–22, which show neutralization activity and recognize the different antigen epitopes. Antibody fragments, including Fab and scFv, have superiority in properties, such as low immunogenicity, high infiltration, and a low cost, and may be therapeutic candidates instead of intact antibodies [23,24]. Since there is a report that the domain orders of the VH and VL affect the productivity and biological activity of scFvs [25,26], we designed both scFv HL and scFv LH constructs for each mAb. All the scFvs were prepared with high purity (Figure 2d,e), even the 15−13 scFv LH, which had the best yield among the four types of the scFvs showing lower yields compared to the previously reported scFv of CR57 [27]. In both the 15–13 and 12–22 scFvs, the scFv LHs were more highly expressed as inclusion bodies and more efficiently refolded than their scFv HLs (Table 3). Both the 12–22 scFv HL and LH tended to aggregate, and their expression levels were lower than those of the 15–13 scFvs (Table 3). We evaluated the thermal stability of these antibody fragments, and the *T*_m_ values of all the scFvs were lower than those of the Fab fragments, suggesting that they are less stable (Figure 4a,b). The difference in the *T*_m_ values between the 15–13 scFv HL and the 15–13 scFv LH was 2.5 °C, indicating that the scFv LH, which had more refolding efficiency, showed higher stability. In addition, the RFU of the DSF plot at 20 °C showed that the values of the scFv HL were higher than those of the scFv LH and Fab fragments for both the 15–13 and 12–22 mAbs. SYPRO orange binds to the hydrophobic region of the protein and increases the fluorescence intensity [28], suggesting that the hydrophobic region is easily exposed in the scFv HL for both the 15–13 and 12–22 mAbs, which might be related to the low refolding efficiency. These results may provide insights into the design and production of stable antibody fragments.

The *K*_D_ values of the 15–13 scFv HL and the 15–13 scFv LH to the rRABVG were determined to be 24.9 nM and 17.7 nM, respectively, exhibiting high binding affinity, although slightly worse than that of the 15–13 Fab (*K*_D_ = 5.6 nM) (Figure 5d). Both the scFvs showed slower association and faster dissociation than the Fab, which may be due to the lack of domain–domain interaction by the CH1 and CL, resulting in the decreasing stability of the fragments. However, the *K*_D_ value of the CR57 and CR4098 mAbs (whole antibody), which comprises the CL184 cocktail, was 2.4 nM and 4.5 nM, respectively [29], suggesting that the 15–13 antibody fragments showed slightly lower, but comparable, binding activity to these antibodies. We performed the neutralization activity assay, and the preliminary experiments of the infectious assay using the rabies virus showed that both scFvs have neutralization activity. On the other hand, the 12–22 fragments showed lower a binding affinity (*K*_D_ ~ μM) than the 15–13 fragments (Figure 6a-d**)**. The kinetic analysis demonstrated that all of the 12–22 fragments tended to associate slower and dissociate faster than the 15–13 fragments. However, the preliminary experiments of infection inhibition showed that these fragments exhibit neutralizing activity. Therefore, these antibody fragments are suggested to be potential anti-RABV biopharmaceutical candidates. Furthermore, these results also suggest that, since the constant regions (CH1-CH2-CH3, and CL1) for the 15–13 and 12–22 antibodies had no effect on the binding to the RABVG and on the inhibition of the RABV infection, their Fvs should recognize the receptor binding site and/or its close area. Currently, structural information for the RABVG complex of these scFvs is being investigated by cryo-electron microscopy, which will further provide insight into the RABVG binding modes and the design of the humanization and antibody cocktails for these antibodies.

## Figures and Tables

**Table 1:** Information on primers used for scFv construction. Bold text indicates restriction sites, and underlines indicate the GGGGS repeat linker.

Name	Sequence
For 15–13	
15–13 scFv HL_HF	5′- GGGATTC**CATATG**GAGGTGAAGGTGGTGGAGTC -3′
15–13 scFv HL_HR	5′- CTGCCGCCACCACCAGACCCACCGCCGCCTGAGGAGACGGTGACTGAG -3′
15–13 scFv HL_LF	5′- GGTGGTGGCGGCAGTGGAGGTGGTGGATCCGATATTGTTTTGTCACAGTCTCCAGC -3′
15–13 scFv HL_LR	5′- GGC**GAATT**CTCATTTGATTTCCAGCTTTGTCCCAG -3′
15–13 scFv LH_LF	5′- GGGATTC**CATATG**GATATTGTTTTGTCACAGTCTC -3′
15–13 scFv LH_LR	5′- CTGCCGCCACCACCAGACCCACCGCCGCCTTTGATTTCCAGCTTTGTC -3′
15–13 scFv LH_HF	5′- GGTGGTGGCGGCAGTGGAGGTGGTGGATCCGAGGTGAAGGTGGTGGAGTC -3′
15–13 scFv LH_HR	5′- GGC**GAATTC**TCATGAGGAGACGGTGACTGAG -3′
For 12–22	
12–22 scFv HL_HF	5′- GGGATTC**CATATG**GAAGTGCAACTGGTGGAGTCTG -3′
12–22 scFv HL_HR	5′- CTGCCGCCACCACCAGACCCACCGCCGCCCGAGGAAACGGTGACCG -3′
12–22 scFv HL_LF	5′- GGTGGTGGCGGCAGTGGAGGTGGTGGATCCGATATTGTGTTATCACAGTCTCCAGCC -3′
12–22 scFv HL_LR	5′- GGC**GAATTC**TCATTTTATTTCCAGCTTGGTCCCAG -3′
12–22 scFv LH_LF	5′- GGGATTC**CATATG**GATATTGTGTTATCACAGTCTC -3′
12–22 scFv LH_LR	5′- CTGCCGCCACCACCAGACCCACCGCCGCCTTTTATTTCCAGCTTGG -3′
12–22 scFv LH_HF	5′- GGTGGTGGCGGCAGTGGAGGTGGTGGATCCGAAGTGCAACTGGTGGAGTCTG -3′
12–22 scFv LH_HR	5′- GGC**GAATTC**TCACGAGGAAACGGTGACCGTG -3′

**Table 2:** Information on primers used for RABVG construction: Bold text indicates restriction sites, and underlines indicate mutation sites.

Name	Sequence
For RABVG	
RABVG_ECD_F	5′- **CTAGCTAGCTTCCCCATTTACACGATACC** -3′
RABVG_ECD_R	5′- CCG**CTCGAG**TGATTTCGGGAGACC -3′
pFastBac1_F	5′- CCTATAAATATTCCGGATTA-3′
RABVG_ECDm_FL1F	5′- GGAGGATCTGGAGGAACCACATTCAAGAG -3′
RABVG_ECDm_FL1R	5′- TCCTCCAGATCCTCCGGTGTAGGTCTCTGC -3′
RABVG_ECDm_FL2F	5′- GGTGGTAGTGGTGGTAGAACCACCAAAGAG -3′
RABVG_ECDm_FL2R	5′- ACCACCACTACCACCGTATGGATTGTGTAG -3′
pFastBac1_R	5′- CAAATGTGGTATGGCTGATT-3′

**Table 3:** Expression and preparation efficiency of each scFvs. Inclusion body and final yields from 1L of culture medium are shown.

Name	Inclusion Body (mg/L)	Refolding Efficiency (%)	Final Yields (mg/L)
15–13 scFv HL	43.6	12.2	5.3
15–13 scFv LH	85.8	14.5	12.4
12–22 scFv HL	57.4	1.4	0.8
12–22 scFv LH	73.6	3.0	2.2

## Data Availability

The data supporting the findings of this study are available from the corresponding author, K.M., upon reasonable request.
